# A systematic review of concepts related to women’s empowerment in the perinatal period and their associations with perinatal depressive symptoms and premature birth

**DOI:** 10.1186/s12884-017-1495-1

**Published:** 2017-11-08

**Authors:** Esmeralda R. Garcia, Ilona S. Yim

**Affiliations:** Department of Psychology and Social Behavior, University of California, 4562 Social and Behavioral Sciences Gateway, Irvine, CA 92697-7085 USA

**Keywords:** Empowerment, Perinatal depression, Preterm birth, Low birthweight, Prematurity

## Abstract

**Background:**

The perinatal period, which we here define as pregnancy and the first year postpartum, is a time in women’s lives that involves significant physiological and psychosocial change and adjustment, including changes in their social status and decision-making power. Supporting women’s empowerment at this particular time in their lives may be an attractive opportunity to create benefits for maternal and infant health outcomes such as reductions in perinatal depressive symptoms and premature birth rates. Thus, we here systematically review and critically discuss the literature that investigates the effects of empowerment, empowerment-related concepts and empowerment interventions on reductions in perinatal depressive symptoms, preterm birth (PTB), and low birthweight (LBW).

**Methods:**

For this systematic review, we conducted a literature search in PsychInfo, PubMed, and CINAHL without setting limits for date of publication, language, study design, or maternal age. The search resulted in 27 articles reporting on 25 independent studies including a total of 17,795 women.

**Results:**

The majority of studies found that, for the most part, measures of empowerment and interventions supporting empowerment are associated with reduced perinatal depressive symptoms and PTB/LBW rates. However, findings are equivocal and a small portion of studies found no significant association between empowerment-related concepts and perinatal depressive symptoms and PTB or LBW.

**Conclusion:**

This small body of work suggests, for the most part, that empowerment-related concepts may be protective for perinatal depressive symptoms and PTB/LBW. We recommend that future theory-driven and integrative work should include an assessment of different facets of empowerment, obtain direct measures of empowerment, and address the relevance of important confounders, including for example, ethnicity and socioeconomic status.

**Electronic supplementary material:**

The online version of this article (doi:10.1186/s12884-017-1495-1) contains supplementary material, which is available to authorized users.

## Background

Women’s empowerment can lead to significant positive changes in many domains. In terms of health, studies have found an association between increased empowerment and reduced mortality and morbidity [1, 2]. For example, empowerment interventions have been associated with decreases in blood glucose and cholesterol levels among women with pre-diabetes and high cardiovascular disease risk [3]. In terms of reproductive health, empowerment has been associated with reduced rates of unintended pregnancies [4] and sexually transmitted diseases, such as chlamydia and gonorrhea, in high-risk populations [5]. Other studies have shown benefits of empowerment for health-related behaviors such as obtaining nutritional supplements and participating in health education sessions [6].

The benefits of empowerment are not necessarily limited to women themselves, but have the potential to extend to those around her, including – perhaps most prominently – her own children. Stressors experienced during pregnancy not only result in physiological alterations in the pregnant woman, but these biological signals can be communicated to the unborn child via placental transmission, and have been associated with outcomes including preterm birth (PTB) and postpartum depression [7, 8].

The perinatal period, here defined as pregnancy and the first year postpartum, is also a time in women’s lives that involves significant physiological and psychosocial change and adjustment, including changes in women’s social status and decision-making power. Supporting women’s empowerment at this particular time may be an opportunity to create long-lasting benefits, not only for the new mother, but also for her newborn. One of the earliest measures of infant health are measures of prematurity including PTB and low birthweight (LBW), and there is convincing evidence that being born prematurely poses a risk factor for poorer health outcomes throughout the life span (e.g., [9]). In terms of maternal health, an early birth outcome is the presence and degree of postpartum depressive symptoms, which in the broader postpartum depression literature have been assessed to occur anywhere between 1 day and 1 year postpartum [10]. It has been argued that the bio-behavioral pathways leading to PTB and postpartum depression and its symptoms may overlap [11], which led us to include both health outcomes in this review.

Herein, we systematically review the literature testing the link between perinatal maternal empowerment and perinatal depressive symptoms as well as PTB and LBW. Because the number of studies testing these associations is very small, we chose to define empowerment in its broadest sense as a person’s autonomy, decision-making power, and self-determination. We also decided to include studies on empowerment-related concepts that merely relate to or impact on empowerment if the authors’ discussion of the methods used fell within the framework of empowerment. Conceptualizations of empowerment and its related concepts among studies in this review include, for example, relationship power, equity, self-efficacy reflected in women’s financial independence, reductions in intimate partner violence, and increases in domestic decision-making power.

The present review includes observational studies assessing the degree of empowerment and empowerment-related concepts through questionnaires, as well as studies supporting women’s empowerment by implementing programs intended to increase women’s empowerment, which we here refer to as empowerment interventions. Of note, among the intervention studies, only one actually measured changes in an empowerment-related concept [12]. Moreover, the interventions in the studies reported here were not always designed to improve a health outcome by changes in empowerment alone. Table 1 shows which measure or intervention was used for each study, and how empowerment was conceptualized.

**Table 1:** Table of concepts

Measure of empowerment or intervention	Empowerment components		References
	Intervention	Direct measure	
INTERVENTION STUDIES			
CenteringPregnancy (CP)	Self-efficacy, self-care, self-esteem, knowledge		[15, 17, 24, 25, 29, 33–41]
Parent-to-parent and parent-to-provider dialogue	Self-efficacy, partnership, participation, collaboration, awareness, sense of control, self-help, meeting personal needs, access to resources, and personal action		[18]
Guided Participation	Self-confidence, self-efficacy		[22]
Health locus of control	Internal health locus of control, self-efficacy, health knowledge	Health locus of control	[12]
Mom Power	Self-confidence, parenting competence, self-efficacy, knowledge, self-care		[23]
Creating Opportunity for Parent Empowerment (COPE)	Parenting confidence and knowledge, participation in care, self-confidence, control		[26–28]
OBSERVATIONAL STUDIES			
Gender hierarchies within the family, unequal power relationships, domestic decision-making power		Agency, decision-making power	[14]
Locus of control		Internal locus of control, self- efficacy, self-competence	[16]
Intimate partner violence, relationship power, and equity		Relationship power	[20]
Domestic violence and not having the right to plan pregnancy		Agency, decision-making power	[30]
Debt and lack of financial decision-making power		Agency, decision-making power	[31]
Domestic decision-making power		Agency, decision-making power	[32]

## Methods

### Search strategy

We conducted a literature search in PsychINFO, PubMed, and CINAHL, according to guidelines in the PRISMA (Preferred Reporting Items for Systematic Reviews and Meta-Analyses) statement [13]. The terms ‘agency’, ‘autonomy’, ‘choice’, ‘control’, ‘domestic decision-making power’, ‘economy’, ‘empower’, ‘empowerment’, ‘fertility intervention’, ‘leadership’, ‘power’, ‘pregnancy’, ‘resources’, ‘transformation’, ‘voice’, and ‘women’s health’ were combined with the search terms ‘birth’, ‘birthweight’, ‘preterm’, ‘depression in pregnancy’, ‘perinatal depression’, ‘postpartum depression’, and ‘premature birth’. The search terms ‘CenteringPregnancy’, ‘child marriage’, and ‘sexual activity’ also emerged as relevant due to their presence in the initial search results, and the three terms were thus added to the final search.

### Inclusion and exclusion criteria

To be included in this review, studies had to be peer-reviewed and include (1) a sample of women who were either pregnant or within the first year postpartum; (2) a measure of empowerment or lack thereof or an intervention aimed at supporting women’s empowerment; and (3) a measure of perinatal depressive symptoms or of prematurity (PTB, LBW). Of note, whereas PTB/LBW is determined at the time of birth, maternal depressive symptoms can occur in the postpartum period as well. Thus, studies of empowerment in the first year postpartum were only relevant to the literature on depressive symptoms. We identified 1 study assessing depressive symptoms during pregnancy, 11 in the postpartum period, and 4 in both pregnancy and postpartum. Empowerment was measured or an intervention supporting empowerment was administered postpartum for 9 studies, in pregnancy for 3 studies, and in both for 4 studies. No limits were set for date of publication, maternal age, study design, or language of publication; nevertheless, only English language publications were identified.

### Selected studies

**Fig. 1:**
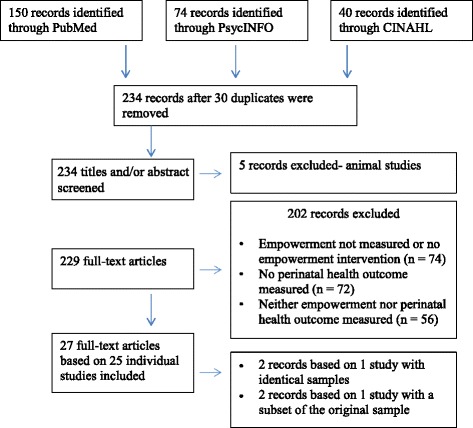
Flowchart following guidelines in the PRISMA (Preferred Reporting Items for Systematic Reviews and Meta-Analyses) statement [13]

The literature search identified 150 records in PubMed, 74 records in PsycINFO and 40 records in CINAHL (see Fig. 1 for PRISMA flow chart). After removing 30 duplicates, 234 articles were screened for eligibility. Five records were excluded based on title and abstract reviews because they were animal studies. Of the remaining 229 full-text articles, 202 records were excluded because they did not include a measure of empowerment or an intervention supporting empowerment (n = 74); did not measure perinatal depressive symptoms, PTB, or LBW (n = 72); or lacked a measure of empowerment as well as perinatal depressive symptoms and PTB or LBW (n = 56). The remaining 27 articles were included in this manuscript. These articles report on 25 independent studies with a total of 17,795 participants. Across studies, sample sizes ranged from 30 [14] to 6155 [15], with a mean of 693 participants (SD = 1196). Most studies were conducted in the US (70.83%), and the remaining studies in Iran, Pakistan (both 8.33%), Taiwan, Canada, and the Netherlands (all 4.17%). Of the studies conducted in the US, one did not provide ethnicity information [16]. In the remaining studies, the majority of participants were African-American (36.27%), followed by White (28.13%), Hispanic (24.42%), Asian (7.01%), Native American (<1%), and Other (4%). Average participant ages ranged from 15 [17] to 33 years old [18]. Across studies, participants were an average of 25.01 years old (SD = 3.79).

### Quality ratings

Study quality ratings were conducted according to National Institute of Health standards for controlled intervention studies as well as for observational, cohort, and cross-sectional studies [19]. We considered studies with a quality score of eight to 14 of good quality, and those with scores of five to seven as fair. While the National Institute of Health standards recommend to not base quality ratings on sum scores alone, we contend that this categorization also matched our individual readings of each study. No studies were considered to be of poor quality.

### Conceptualizations of empowerment

We identified six studies that assessed empowerment in cross-sectional/observational study designs and 19 studies that reported on empowerment interventions. Observational studies used mostly self-report or interview measures, such as the Decision-Making Dominance Subscale of the Sexual Relationship Power Scale [20] and critical ethnographic interviews [14]. Studies used different types of empowerment interventions (see Table 1 for details). The majority report on CenteringPregnancy (CP), a group prenatal care model. “*CenteringPregnancy unifies the components of prenatal care—risk assessment, education, and support within the group—and encourages women to take responsibility for their own health*” ([21], p. 46). CP was included in this review because it aims to empower women by increasing their health self-care efficacy during pregnancy. “*The woman’s involvement in self-care activities, the discussion and education format, the worksheets and handouts, and the sharing among the women all lead to her enhanced sense of empowerment. This, in turn, results in a sharing of power between the provider and the consumer*” ([21], p. 53). Other studies report on the Creating Opportunities for Parent Empowerment (COPE) program for parents with premature infants in Neonatal Intensive Care Units. COPE aims to inform parental decision-making and increase parental knowledge about infant developmental outcomes, consequently easing parent–infant interactions. Thus, it aims to support women’s empowerment by increas[es]ing parenting confidence, participation in care, self-confidence, and control, affecting health outcomes including reducing parenting anxiety and depressive symptoms as well as reducing their stay in the Neonatal Intensive Care Units.

The remaining studies used various other interventions. The first is the Guided Participation program. It is included in the framework of empowerment-related models because it acts on autonomy and self-determination by informing mothers of premature infants of effective premature infant care and increases parenting self-efficacy, feeding competencies, and positive infant behaviors to reduce anxiety and depressive symptoms associated with parenting a premature infant [22]. This has empowerment implications in terms of promoting self-confidence among mothers of premature infants who may feel helpless, scared, and might lack confidence in their ability to care for their premature newborn. The second is an internal health locus of control intervention that aims to promote empowerment of women through education about self-care such as pregnancy stage monitoring, nutrition, and health [12]. One study reports on a parent-to-parent and parent-to-provider dialogue intervention related to participation, collaboration, awareness, sense of control, self-efficacy, and self-help as well as access to resources and personal action to shift the personal health decision-making power from healthcare provider to client [18]. Finally, Mom Power is a parenting and self-care skills group program for high-risk mothers and their young children that aims to empower by increasing parenting and self-care knowledge [23].

## Results

Of the 27 manuscripts included in this review, 16 report on maternal perinatal depressive symptoms and 11 studies report on PTB or LBW. No studies that addressed both maternal and infant outcomes were identified, and we therefore report on the two types of outcomes separately.

### Maternal perinatal depressive symptoms

Of the 16 studies reporting on perinatal depressive symptoms, 10 were intervention studies and 6 were observational studies (Table 2); 15 studies assessed depressive symptoms postpartum and 12 yielded significant findings (80%), whereas only 2 out of 5 studies (40%) yielded significance when depressive symptoms were assessed in pregnancy. Nine out of 11 studies rated as good (82%) and 4 out of 5 studies rated as fair (80%) yielded significance.

**Table 2:** Articles reporting on empowerment and perinatal depression

Reference ID/Study authors	Study design	Sample	Measure of empowerment/intervention	Measure of depressive symptoms	Major findings	Quality rating/score^a^
INTERVENTION STUDIES						
[18] Liu et al. (2010)	Quasi-experimental	70 parents of preterm newborns in Taiwan, 15 fathers, 55 mothers, *M* age = Intervention: 33.0 years, Control: 32.5 years	Parent-to-parent and parent-to-healthcare provider dialogue PP: 12 sessions over 8 months	BDI-II PP: pre- and post-intervention	Participating in dialogue w/lower post-intervention PPD symptoms, greater pre to post reduction in PPD symptoms, and greater childrearing self-efficacy	Fair (6)
[22] Pridham et al. (2005)	Randomized controlled trial	42 US mother-premature infant pairs, 28 weeks post-conception, *M* age = GP: 25.5 years, Control: 26.17 years; predominantly European American and African American	GP – program to increase feeding competencies PP: weekly, biweekly, or monthly in first year	CES-D PP: 1, 4, 8, 12 months	No difference in PD symptoms; GP w/better ability to regulate infant negative affect and feeding behaviors	Good (9)
[12] Monthshki et al. (2013)	Randomized controlled trial	230 women in Iran; 28–30 weeks’ GA, *M* age = Intervention: 28.0 years, Control: 27.8 years	Multidimensional Health Locus of Control PREG: 28–30 weeks’ GA PP: 4 weeks	EPDS PP: 4 weeks PP	Intervention w/increased internal health locus of control and lower PPD symptoms	Fair (7)
[23] Muzik et al. (2015)	Prospective cohort study	80 US mother-child pairs *M* age = 23.7 years; Ethn: Caucasian 48.4%, African-American 44.1%, Biracial/Hispanic 7.5%	MP – parenting and self-care skills group program for high-risk mothers and their children PP: 13-weeks/sessions	PDSS PP: pre- and post-intervention	MP w/reduction in depression, post-traumatic stress disorder, and caregiving helplessness	Good (12)
[24] Ickovics et al. (2011)	Randomized controlled trial	1047 US pregnant women, ages 14–25 (*M* = 20.4) years, 18 weeks’ GA; Ethn: 80% African-American, 13% Latina, 6% White, 1% Other; Note: Sample identical to [34], in Table 3	CP, CP+ group w/additional HIV prevention information in last 3 sessions PREG and PP	CES-D PREG: 2nd and 3rd trimester PP: 6, 12 months	Initial high stress and CP+ group w/less PPD symptoms 1 year PP	Good (11)
[25] Kennedy et al. (2011)	Randomized controlled trial	322 US pregnant women in the military, 12–16 weeks’ GA; *M* age = Control 25.5 years, CP: 24.0 years; Predominantly White, African American or Latina	CP PREG: 9 group sessions PP: one session	CES-D; PDSS (PP only) PREG: baseline, 32–36 weeks’ GA PP: 3–4 months	No differences in PD and PPD symptoms w/CP	Good (10)
[26] Melnyk et al. (2001)	Randomized controlled trial	42 US mothers of premature infants, *M* age = COPE 26.6 years, Control, 28.8 years; Predominantly White or African American	COPE PP: 2–4 days after admission, 2–4 days thereafter, 1–2 days in NICU, 1–4 days before discharge, 7 days after discharge, 3 and 6 months	POMS PP: at all phases and follow-up	COPE w/less depressive symptoms after admission and before discharge, and w/higher infant cognitive development scores; No difference in mother’s overall mood state	Good (11)
[27] Melnyk et al. (2006)	Randomized controlled trial	260 US families; 258 mothers (*M* age = 27.8 years) and 154 fathers with infant born at 26–34 weeks’ GA; Predominantly White or Black	COPE PP: 2–4 days after admission 2–4 days thereafter, 1–2 days in NICU, 1–4 days before discharge, 7 days thereafter	BDI-II PP: at all times except NICU	COPE w/lower post-hospital parental stress and depressive symptoms	Good (13)
[28] Melnyk et al. (2008)	Randomized controlled trial	246 US mothers of LBW preterm infants, *M* age = 27.9 years, born at 26–34 (*M* = 31.4) weeks’ GA; Predominantly White or Black Note: Sample a subset of [27]	COPE PP: 2–4 days after admission, 2–4 days thereafter, 1–2 days in NICU, 1–4 days before discharge, 7 days thereafter	BDI-II PP: 2–4 days after NICU admission, 2–4 days thereafter, and 2 months post-intervention	COPE w/decreased post-hospital depression and anxiety symptoms	Good (13)
[29] Robertson et al. (2009)	Quasi-experimental	49 Hispanic women, 24–26 weeks’ GA, *M* age = Control: 26.5 years, CP: 24.6 years	CP PREG: throughout pregnancy	CES-D PP: not specified	No difference in PD symptoms w/CP	Fair (5)
OBSERVATIONAL STUDIES						
[14] O’Mahony et al. (2013)	Qualitative	30 non-European immigrant and refugee women < 18 years old, living in Canada < 10 years, high risk for PPD	Relationship dominance and control, at time of interview PP: weeks not specified	EPDS PREG and PP: within past 5 years and again at time of interview	Emotional and economic relationship dominance by partner w/greater self-reported PPD vulnerability and symptoms	Fair (6)
[16] Richardson et al. (2012)	Cross-sectional	126 US rural pregnant women, 18–50 years, mostly Caucasian	Levenson Scale on Locus of Control PREG: 20–28 weeks’ GA	EPDS PREG: 20–28 weeks’ GA	Higher external locus of control w/higher PD scores; Internal locus of control w/lower PD	Fair (6)
[20] Gibson et al. (2015)	Cross- Sectional	182 US pregnant and PP women, *M* age = 18.8 years; Ethn: 43% Hispanic, 38% African American, 15% White, 4% Other	Intimate partner violence w/CTS, Partner power w/Decision-Making Dominance Subscale of the SRPS PREG: 2nd or 3rd trimester PP: 6 months	CES-D PP: 6 months	PPD symptoms w/higher partner power and intimate partner violence	Good (9)
[30] Ali et al. (2009)	Longitudinal	420 Pakistani pregnant women, *M* age = 26.1 years, 27.1% local, 72.9% immigrants	Unplanned pregnancy and domestic violence questionnaires PP: within 10 weeks pp	Aga Khan University Anxiety and Depression Scale, DSM IV PP: consent, 1, 2, 6, 12 months	Domestic violence and unplanned pregnancy w/higher PPD risk	Good (11)
[31] Rahman et al. (2012)	Longitudinal	791 rural Pakistani women, *M* age = 26.8 years	Empowered to manage household finances: yes/no PREG: 3rd trimester PP: 6 months	SCID, HRSD PREG: 3rd trimester PP: 6 months, 1 year	Household debt and lack of empowerment to manage household finances w/PD and PPD	Good (11)
[32] Chien et al. (2012)	Cross-sectional	380 immigrant women (*M* age = 27.06) from China and Vietnam, and native women (*M* age = 31.7) in Taiwan	Domestic decision-making power scale PP: 1–12 months	EPDS PP: 1–12 months	Low domestic decision-making power, family income, low social support and immigrant status w/higher PPD symptoms	Good (8)

^a^Quality rating score is number of criteria met according to the National Institute of Health quality rating scale (range 0–14)

Studies are listed in order of their Reference Section ID Number

*BDI-II* Beck Depression Inventory-II, *CES-D* Center for Epidemiological Studies Depression Scale, *COPE* Creating Opportunities for Parent Empowerment, *CP* CenteringPregnancy, *CTS* Conflict Tactics Scale, *EPDS* Edinburg Postnatal Depression scale, *Ethn* Ethnicity, *GA* gestational age, *GP* guided participation, *HRSD* Hamilton Rating Scale for Depression, *LBW* low birthweight, *M* Mean, *MP* Mom Power, *NICU* neonatal infant care unit, *PD* perinatal depression, *PDSS* Postpartum Depression Screening Scale, *POMS* Profile of Mood States Scale, *PP* postpartum, *PPD* postpartum depression, *PREG* pregnancy, *SCID* Structured Clinical Interview for Depression, *SRPS* Sexual Relationship Power Scale, *US* United States, *w/* with

### Intervention studies

Among the intervention studies were seven randomized controlled trials [12, 22, 24–28], two quasi-experimental studies [18, 29], and one prospective cohort study [23].

Three of the randomized controlled trials, all by the same team of authors, used the COPE program as an experimental intervention. The first of this set of studies evaluated COPE in a small sample of 42 mothers of premature infants [26]. When depressive symptoms were assessed 4 to 8 days after admission and 1 to 4 days prior to discharge, women receiving the intervention had fewer depressive symptoms than those receiving traditional care. However, no differences were observed when scores were aggregated across all phases of the intervention. A second study of 260 families confirms that mothers in COPE report fewer post-hospital depressive symptoms than those in the control group, and further expands by suggesting that COPE may also reduce parental stress [27]. The last article reporting on a subset of 246 mothers found participation in COPE was related not only to mothers’ decreased post-hospital depressive symptoms, but also to reduced anxiety [28]. Thus, while COPE was conceptualized to improve premature infant health and developmental outcomes, the findings of these three studies suggest that COPE may also be an effective tool for reducing maternal postpartum symptoms of depression, stress, and anxiety.

Two studies reported on CP randomized controlled trials [24, 25]. Among women who initially reported high stress, those in a ‘CP+’ group, which added information about HIV prevention as well as components of psychosocial functioning such as behavioral risk assessment, goal setting, communication, and negotiation skills, had significantly fewer depressive symptoms at 1 year postpartum than those in the standard CP and control groups [24]. These findings may suggest that CP could be useful in terms of improving maternal depressive symptoms if it also addresses the specific needs of the patient population. Another CP study of 322 pregnant women in the military found no differences in prenatal or postnatal depressive symptoms between those in a CP condition and those receiving standard care [25]. Similarly, a smaller quasi-experimental study of 49 Hispanic women suggests no significant differences in perinatal depressive symptoms between those in CP and those receiving traditional care [29]. However, in addition to the small sample, the study groups differed in that those in the CP group had more primigravidas.

Of the remaining studies, three found significant associations between empowerment and perinatal depressive symptoms. The strongest support comes from a prospective cohort study of 80 mother–child pairs who participated in the Mom Power intervention. Results suggest that participation in Mom Power is not only associated with significant reduction in depressive symptoms, but also with lower symptoms indicative of post-traumatic stress disorder risk and reduced caregiving helplessness [23]. Another study empowered women by providing an internal health locus of control intervention and found significant increases in internal health locus of control as well as lower postpartum depressive symptoms among those in the intervention group [12]. Similarly, a study of 70 Taiwanese parents of preterm infants, using a parent-to-parent and parent-to-provider dialogue, found that post-intervention depression scores were significantly lower for those in the intervention than for those in the control group. Those in the intervention group also had higher childrearing self-efficacy [18]. In contrast, findings from a small study of 42 mothers with very LBW infants (≤1200 g) indicate no differences in maternal perinatal depressive symptoms between those in the Guided Participation and control groups [22]. However, those in the intervention group were better able to regulate infant negative affect and feeding behaviors than those in the control group.

### Observational studies

Among the 6 observational studies, 2 were longitudinal [30, 31], 3 cross-sectional [16, 20, 32], and 1 used a qualitative study design [14]. Despite variations in definitions of empowerment, all quantitative studies yielded significance, and studies suggest a positive association between perinatal depressive symptoms and domestic violence [20, 30], emotional and economic relationship dominance by an intimate partner [14], and external locus of control [16] as well as negative associations with domestic decision-making power [32] and the power to manage household finances [31].

### Summary

In sum, all observational and all but three of the intervention studies [22, 25, 29] suggest reduced perinatal depressive symptoms with empowerment. More specifically, studies using the COPE, Mom Power, internal health locus of control, parent-to-parent and parent-to-provider dialogue interventions were effective in reducing maternal perinatal depressive symptoms. In contrast, the CP intervention did not significantly improve maternal affect, unless adaptations specific to the study population were made. Similarly, the Guided Participation intervention did not improve maternal health outcomes, likely because it was not primarily developed to address maternal health outcomes, but to improve infant feeding competencies and affect regulation.

### PTB and LBW

All 11 studies on PTB and LBW were intervention studies and all used the CP intervention (Table 3). Of these, one was a prospective cohort study [33], two were randomized controlled trials [34, 35], and eight were retrospective cohort studies [15, 17, 36–41]. The prospective cohort study [33] reports that mothers in CP had infants with higher birth weight and longer gestational age at birth than those in the control group. Moreover, among those who delivered preterm, the CP group participants maintained gestation for significantly longer. Similarly, a randomized controlled trial of 1148 women found reduced rates of PTB and LBW among women in the CP+ group [35]. A second randomized controlled trial of 1047 women reports that those in the CP group were significantly less likely to have PTB in comparison to the control group, but no significant group differences were found for LBW [34]. This study also reports a dose–response effect such that gestational age decreased in relation to the number of group sessions missed, suggesting the null findings may be attributable to factors other than lack of effectiveness of the intervention. All of the studies were rated as good quality, ranging from 9 to 12, but studies that found no significance tended to be those at the lower end of the good quality range, receiving scores of 9 and 10.

**Table 3:** Articles reporting on empowerment and preterm birth/low birthweight

Reference ID/Study authors	Study design (all are interventions)	Sample	Measure of empowerment/intervention	Measure of PTB/LBW	Major findings	Quality rating/score^a^
[15] Tanner-Smith et al. (2014)	Retrospective cohort study	6155 US pregnant women, *M* age = 25.0 years; African American 49.9%, White 30.3%, Hispanic 20.7%	CP, throughout pregnancy	MR	CP w/longer gestation, higher birth weight, lower odds of very LBW, but not LBW or PTB	Good (12)
[17] Grady et al. (2004)	Retrospective cohort study	124 US pregnant adolescents, *M* age = CP: 15.8 years, 2001 comparison: 16.5 years, 1998 comparison: 16.3 years; 12–18 weeks’ GA; Predominantly African American	CP, 12–18 weeks’ GA, and every 2 weeks until 8 weeks postpartum	MR	CP group w/less LBW and PTB	Good (11)
[33] Ickovics et al. (2003)	Prospective cohort study	458 US pregnant women, 12–40 years (*M* = 21.6); ≤ 24 weeks’ GA; African American 80%, Latina 15%, and White 5%	CP, throughout pregnancy	MR	CP w/higher birth weight and longer gestation, but not PTB or LBW	Good (11)
[34] Ickovics et al. (2007)	Randomized controlled trial	1047 US pregnant women, 14–25 years (*M* = 20.4); 18 weeks’ GA; Predominantly African American and Latina	CP, throughout pregnancy	MR	CP group w/lower likelihood of PTB, but not LBW	Good (11)
[35] Ickovics et al. (2016)	Randomized controlled trial	1148 US pregnant women, 14–21 years; < 24 weeks’ GA; Predominantly Latina, Black, White	CP+, throughout pregnancy	MR	Greater number of CP visits w/lower odds of small for GA neonate, PTB, and LBW	Good (11)
[36] Barr et al. (2011)	Retrospective cohort study	379 US pregnant women, *M* age CP: 27.1 years, Control: 27.4 years; Predominantly Hispanic, Black, White	CP, in pregnancy; GA not specified	MR	CP w/lower odds of PTB and a trend toward less LBW compared to standard care	Good (9)
[37] Klima et al. (2009)	Retrospective cohort study	268 US pregnant women, ages 14–38 (21.8) years, ≤ 18 weeks’ GA; 100% African American	CP, throughout pregnancy	MR	No difference in PTB and LBW w/CP	Good (10)
[38] Picklesimer et al. (2012)	Retrospective cohort study	4083 US pregnant women, *M* age = 23.1 years, ≤ 16 weeks’ GA; Predominantly White, Black or Hispanic	CP, throughout pregnancy	MR	CP w/less PTB, but not LBW	Good (11)
[39] Tandon et al. (2012)	Retrospective cohort study	216 US pregnant women, *M* age = CP: 27.4 years, Control: 27.5 years; ≤ 20 weeks’ GA; 100% Hispanic/Mayan	CP, throughout pregnancy	MR	CP w/lower PTB, but not LBW	Good (10)
[40] Trudnack et al. (2013)	Retrospective cohort study	487 US pregnant women, M age = 25.6 years; 100% Latinas	CP, throughout pregnancy	MR	No difference in LBW or PTB w/CP	Good (9)
[41] Walton et al. (2015)	Retrospective cohort study	404 US pregnant women in the military, *M* age = CP: 24.8 years, Control: 26.3 years; Predominantly Caucasian, Asian, African American	CP, throughout pregnancy	MR	No significant difference in LBW and PTB w/CP	Good (9)

^a^Quality rating score is number of criteria met according to the National Institute of Health quality rating scale (range 0–14)

Studies are listed in order of their Reference Section ID Number

*CP* CenteringPregnancy, *GA* gestational age, *LBW* low birthweight, *M* [median]mean, *MR* medical records, *PTB* preterm birth, *US* United States, *w/* with

Among the eight retrospective cohort studies, five provided evidence of at least some associations between empowerment and prematurity. One of these studies found CP to be associated with lower PTB and LBW rates in a sample of 124 adolescents [17]. Three studies, including samples of 216 Latinas [39] as well as ethnically diverse samples of 4083 [38] and 379 [36] women, had mixed findings, reporting reductions in the rate of PTB but not of LBW. A final study reports higher birth weight and higher gestational age at birth as well as significantly reduced risk of very LBW in the CP compared to the control group [15].

The remaining three retrospective cohort studies, all with homogenous samples including 268 African-American women [37], 487 Latinas [40], and 404 US military women [41], suggest no impact of CP on either PTB or LBW. These null findings may, at least in part, be explained by confounding variables. For example, one study reports that those in CP were significantly younger than those in standard care [37], and another reports that those in CP were more likely to be in active duty, younger, and primiparous [41], but these variables were not statistically controlled for.

### Summary

Six of the CP studies found significant benefits for PTB and/or LBW, with an additional two studies suggesting benefits for gestational age and birthweight. Specifically, a retrospective cohort study and a randomized controlled trial utilizing the CP+ intervention found significant benefits of CP for both PTB and LBW. An additional four studies found an association between CP and reduced PTB, but not LBW. Similarly, two studies found significantly higher gestational age at birth and birthweight among CP participants compared to those in the control group; however, effects for PTB and LBW did not reach significance. Three other CP studies found no significant association with gestational age, birthweight, PTB, or LBW. Confounds related to sample characteristics and study design may play an important role in the effectiveness of CP.

## Discussion

We set out to systematically review the relatively small literature on empowerment and maternal and infant health. While all studies meeting inclusion criteria conceptualized their measures in the context of empowerment, none of the studies included a measure of empowerment. Instead, facets of empowerment or concepts related to empowerment were assessed in all cross-sectional studies and in one intervention study. The remaining intervention studies did not assess empowerment, limiting the conclusions that can be drawn about the role of maternal empowerment interventions in maternal and infant health. However, it is remarkable that despite the differences in how maternal empowerment was conceptualized across studies, the majority of studies provide fairly consistent evidence for a link between what was conceptualized as maternal perinatal empowerment and reduced perinatal depressive symptoms and PTB/LBW rates.

In determining why, among the intervention studies, some yielded significance whereas others did not, the conceptualization of empowerment appears to play an important role. All studies of empowerment and prematurity, and the majority of studies on maternal perinatal depressive symptoms used CP as an empowerment strategy. Our review suggests that, in the majority of studies, CP was successful at improving infant outcomes. In contrast, CP was not associated with lower maternal perinatal depressive symptoms, unless the intervention was modified to include components specific to the study population (i.e., the CP+ condition; [24]). CP was developed and tested with the goals of increasing birth weight, decreasing prematurity and improving patient and healthcare provider satisfaction [42], but not to address pregnant women’s emotional needs. Thus, it is perhaps not surprising that significant findings were limited to the studies assessing infant outcomes. Nonetheless, the studies demonstrating benefits to maternal wellbeing with modified versions of the CP interventions suggest that CP could be further developed to also provide benefits for maternal perinatal wellbeing.

Although CP was not associated with maternal depressive symptoms, other empowerment interventions seemed to be associated with lower depressive symptoms postpartum. It appears that the successful interventions were those that aimed to provide women with the coping skills for the stressors ahead. For example, the successful COPE intervention was conceptualized to empower parents, through guided education, to cope with the needs of premature infants, reduce parenting anxiety, and increase child care efficacy. Other interventions successful at improving maternal depressive symptoms were the Mom Power program, aimed at emphasizing an internal health locus of control, and the intervention improving parent-to-parent and parent-to-provider dialogue. Similar to COPE, these interventions were developed to improve parental wellbeing. Providing some additional support for a link between women’s empowerment in the perinatal period and improved maternal mental health comes from the six observational studies, all of which yielded at least some evidence of a link between empowerment and maternal mood. In contrast, CP and the Guided Participation Intervention focused on improving infant outcomes instead of maternal emotional needs, which may explain the null findings reported for maternal depressive symptoms.

While the CP intervention seems to be effective in improving infant birth outcomes in most studies, not all studies yielded significance. One possibility is that relevant confounding variables that were not statistically controlled for contributed to these null findings. For example, studies with null findings tended to have significantly more primiparous [41] and younger [37] women in the CP groups. They were also more likely to have rather homogenous samples, such as women in the military, with less ethnic diversity, or samples with a high proportion of disadvantaged or low-income women [37, 40]. Others include only parents of severely LBW infants (≥1250 g), have inconsistent intervention lengths, and report dose–response effects [22, 34], which may have also contributed to the null findings. Variations in social and material disadvantage [43], as well as in perceptions of disadvantage in education, employment, and economic status [44], may explain variations in efficacy of empowerment interventions.

It should be noted that this review did not aim to comprehensively review all possible maternal and infant health benefits that may be associated with women’s empowerment. Instead, it focused on one specialized aspect of this association, specifically, perinatal depressive symptoms and PTB/LBW. This is not to say that empowerment does not affect women’s and children’s lives in many other important ways, but rather that these are some of the earliest health benefits that can be assessed. There is ample evidence that maternal perinatal depressive symptoms continue to have negative consequences for the health and wellbeing of the mother and child. For example, associations have been shown with infant negative affectivity [45], poor mother–infant bonding [46], elevated parenting stress [47], as well as physical and mental illness [48]. Similarly, PTB and LBW have enduring adverse consequences for child health and developmental outcomes such as increased risk for neurodevelopmental disabilities [49], attention difficulties [50], and cardiovascular disease later in life [51]. Given these associations, it appears likely that perinatal empowerment is associated with other health benefits to mother and infant, some of which may also be longer lasting. While important, these studies were considered to be beyond the scope of this review.

Empowerment was not directly measured in any of the intervention studies, with the exception of a single study measuring changes in internal health locus of control, a concept related to empowerment [12]. All interventions included at least an element of empowerment (Table 1), and it seems likely that supporting women’s empowerment was a contributor to the health benefits observed. Nonetheless, it cannot be concluded with confidence that women’s perceptions of empowerment changed in response to the intervention or, if they did, whether an increase in empowerment led to the observed health benefits. There is a need for studies that test whether empowerment is indeed a pathway through which these interventions contribute to improving maternal and infant health outcomes. We recommend that future studies, but in particular intervention studies, administer a direct measure of empowerment before and after the intervention.

### Recommendations for future work

It is encouraging that the overall pattern observed in this small literature review is suggestive of significant associations between maternal empowerment-related concepts, perinatal depressive symptoms, and prematurity. To move this literature forward, both theoretically and in terms of the development of successful interventions, we propose several future directions that seem particularly promising, in our view.

First, while most studies provide evidence for an association between empowerment-related concepts and perinatal depressive symptoms or prematurity, we also identified some studies that report null findings. These inconsistencies could be the result of different definitions of empowerment and of differences in how empowerment was measured or supported through interventions. It is also possible that the divergent findings are the result of differences in sample composition, including, for example, age, ethnicity, and socioeconomic status. Moreover, a portion of the studies reviewed here included relatively small sample sizes and some were underpowered because they were originally designed to investigate outcomes other than PTB/LBW and perinatal depressive symptoms [22, 29]. We recommend that future, adequately powered studies carefully test the role of possible moderators in the link between women’s empowerment, perinatal depressive symptoms and prematurity, and that more detailed attention will be given to methods of measuring or supporting empowerment.

Moreover, we note that existing studies have focused on empowerment as it specifically relates to women’s parental role. While the parental role is perhaps most salient in the context of empowerment of pregnant women and new mothers, other significant life changes occur with the birth of a child, in particular the birth of a first child. Across many cultures, new mothers often scale back on or halt their involvement in non-parental societal roles such as work outside the home, leading to decreases in financial independence as well as changes in social relationships and social status. We could not identify any studies that tested whether the empowerment of pregnant or postpartum women in domains other than the parental one is associated with perinatal depressive symptoms or prematurity. It would be an important contribution to the literature to study the relative impact of different facets of empowerment during the perinatal period on maternal and infant birth outcomes, because this knowledge would provide the groundwork for targeted interventions.

Related to the above, we further observe that studies sometimes use a vague conceptualization of empowerment that may be confounded with other interpersonal processes in group intervention studies. For example, the use of group prenatal care models gives rise to companionship and social support as confounding variables because these group processes are distinct from empowerment, but also may be associated with risk of perinatal depressive symptoms [52, 53]. Moreover, many interventions aimed to empower through provision of information, knowledge, and health literacy, but they did not include a measure of empowerment. Including a measure of empowerment in the intervention studies would allow for the identification of particular components of empowerment, such as increases in self-competence that may be particularly beneficial for reducing perinatal depressive symptoms and PTB or LBW. In observational studies, the operationalization of empowerment is somewhat clearer and includes increases in domestic decision-making power, financial autonomy, and internal locus of control.

Finally, we note that the literature on women’s empowerment and PTB/LBW is distinct from that on empowerment and perinatal depressive symptoms. This may be because interventions either focused on improving infant outcomes or on reducing postpartum depressive symptoms in mothers of infants that were already born premature. However, there is evidence that maternal perinatal depression and prematurity are correlated and may, in fact, share pathophysiological pathways [11, 54, 55]. The role of perinatal empowerment as a factor associated with physiological pathways related to both perinatal depressive symptoms and prematurity risk therefore merits further investigation.

## Conclusion

In conclusion, the empirical evidence is promising and suggests that women’s empowerment and interventions supporting empowerment are associated with reduced perinatal depressive symptoms and PTB or LBW under certain conditions. However, more research is necessary before concrete recommendations can be made. In particular, it is important to improve our understanding of aspects of empowerment that are (1) protective for specific maternal and infant health outcomes, and (2) beneficial across a variety of populations versus specific subgroups of women. Studies testing the effectiveness of specific facets of empowerment on health outcomes and studies further investigating the role of confounding variables, including but not limited to ethnicity and socioeconomic status, would be useful first steps to achieve this goal. A better understanding of the subtleties of the link between empowerment and health will contribute to enhancing the content specificity and efficacy of empowerment interventions. Studies should also be adequately sized to provide sufficient statistical power to accommodate more complex statistical analyses, such as those testing mediational models of how empowerment is substantiated biologically. Doing so would provide the opportunity to uncover biobehavioral mechanisms that may lead to improvement in perinatal depressive symptoms as well as PTB and LBW rates. Given the potential for empowerment in the perinatal period to provide benefits for both maternal and infant health, this topic merits further investigation.

## Additional file

Additional file 1: Open peer review. (PDF 271 kb)

## Abbreviations

LBW: low birthweight
PTB: preterm birth
CP: CenteringPregnancy
COPE: Creating Opportunities for Parent Empowerment
PRISMA: Preferred Reporting Items for Systematic Reviews and Meta-Analyses
