# Supplementary material for: A systematic review of concepts related to women’s empowerment in the perinatal period and their associations with perinatal depressive symptoms and premature birth
Source: BMC Pregnancy Childbirth. 2017 Nov 8;17(Suppl 2):347. doi: 10.1186/s12884-017-1495-1 (PMC5688402; doi:10.1186/s12884-017-1495-1)
Supplement: Additional file 1: — Open peer review. (PDF 271 kb) [file 12884_2017_1495_MOESM1_ESM.pdf]

Review reports

Title: A systematic review of concepts related to women's empowerment in the perinatal period and their associations with perinatal depressive symptoms and premature birth

Reviewer 1: Linda Franck

This manuscript presents the findings of a systematic review of the literature regarding the effects of empowerment interventions in the perinatal period on maternal depression and the incidence of preterm birth (PTB)or low birth weight (LBW) births

Major Compulsory Revisions

1. Title: The title of the paper does not accurately reflect the content of the paper. First, it does not indicate that the paper is a systematic review of the literature. Second, it does not clearly state that the subject is empowerment interventions (rather than descriptive associations of a women's innate level of empowerment). Third, it suggests a broad range of maternal and infant health outcomes were explored, when in fact the review was restricted to three outcomes only.
2. Abstract: The abstract background is problematic as described in more detail in comment #3a-c. The term 'milestone' in line 37 is not an accurate description of the perinatal period. The term 'empowering' in line 46 is more accurately termed "empowerment interventions".
3. Background: The Background should be revised and expanded to address the following issues:
    a. Empowerment and empowerment interventions during pregnancy should be defined clearly at the outset and referenced. The Background should make it obvious why the authors selected the terms in the search strategy
    b. Lines 69-73 are unclear and perhaps hyperbole. Please describe more precisely what "enormous and lasting" impacts on children through to society are achieved by empowerment interventions targeted at women – and what specifically are these powerful interventions and why if so powerful have they not been implemented?
    c. Lines 78-81 are unclear. What type of empowerment interventions are the authors referring to? What about maternal anxiety, parenting self-efficacy, lifestyle or health behaviors might be impacted? The authors should explain why they have specifically focused on maternal depression and PTB/LBW?

4. Methods:

a. The inclusion/exclusion criteria should clarify the criteria regarding study design. The tables indicate that the authors included a wide range of study designs and yet this is not described or justified and no criteria for selection of the studies by design type is provided.
b. The authors specify that empowerment (not defined) had to be measured or manipulated. This suggests cohort studies were included, but that is not made clear (and impacts my earlier comments about Abstract and Background). In addition, the timeframe for the intervention is not made clear. The reader is led to believe that the intervention must be delivered pre-birth in order to have an effect on depression or PTB/LBW. And yet later, the COPE intervention delivered to women with preterm/LBW babies is included. The authors also specify that a health outcome must be measured, but this is not defined, where as elsewhere, the authors specifically focus on the outcomes of depression and PTB/LBW. The inclusion/exclusion criteria with regard to interventions and outcomes needs to be made more clear and specific and provide a well-reasoned justification.
c. Conceptualiztions of Empowerment: This section would be more accurately titled "Interventions". The description of Centering Pregnancy^{TM} is inaccurate. It is a group prenatal care model. The authors should more specifically and precisely describe the empowerment elements of the intervention and provide citations. As noted in 4b, COPE seems an inappropriate intervention to include, given that it is initiated after PTB/LBW. Moreover, the empowerment elements are not clearly stated and referenced. Similar problems with definitions and time points for interventions and outcomes measurement are noted in interventions described in lines 135-145. It appears conceptually inconsistent to include prenatal and postnatal interventions for depression and PTB/LBW in the same review.
d. No quality assessment of the studies is provided and this should be done.
e. Although the authors reference PRISMA, it does not appear that they followed the PRISMA (or any other systematic review guidelines) for their selection of studies and analyses. The paper would be greatly strengthened by following such guidelines.

5. Results/Discussion: Because of the major methodological and conceptual concerns described above, the reviewer cannot adequately comment on the Results and Discussion. Moreover, previous systematic reviews of Centering Pregnancy^{TM} and COPE^{TM} have not been included in the discussion

Level of interest - As written, this manuscript is not acceptable for publication

Quality of written English - Acceptable

Declaration of competing interests - None

Reviewer 2: Susan Meffert

When assessing the work, please consider the following points:

1. Is the question posed by the authors well defined?
While I agree with the authors that the transition from woman to mother (especially with the 1st child), poses a substantial risk to empowerment, however it is defined, I am not sure that they authors clearly define terms beyond that. Specifically, there seem to be a number of assumptions embedded within the research question that should be more clearly defined. First, as many of the empowerment interventions that are discussed in the paper revolve around helping women to care for themselves as mothers and to teach parenting/childcare skills, there seems to be an assumption that the difficulties women experience with empowerment after having a child revolve around their struggle to be mothers. While parenthood is not to be underestimated, in my work, I have observed that the struggle to remain empowered in work outside the home and as an economically productive individual is quite high stakes for new mothers – for example, in our studies with Kenya HIV+ women, having a child often marks the a halt of work outside the home beginning of a period of higher economic dependency on husbands, reducing empowerment and sometimes leading to gender based violence.

One way around this would be to be very specific that you want only to investigate strategies for empowering new mothers in their roles as parents. Then, you would need a very good justification for why that is a key issue on which to focus.

My other concern about the specification of the question is that there seems to be conflation of the terms depression, empowerment, overall health and even PTSD. Depression and PTSD are medical diseases and are not normal responses to stress—even that as severe as first time parenthood. While empowerment is certainly important, it is not an evidence-based treatment for depression or PTSD and it would be misleading (and potentially damaging) to suggest that it is. My suggestion would be either focus VERY specifically on depression, acknowledging nad defining it as the medical disease it is, then explain why the relationship between empowerment and depression is important even though empowerment is not an evidence based treatment for depression (e.g., you might look at risk factors/prevention).

2. Are the methods appropriate and well described?
Yes, although I would suggest re-organization such that there are not two distinct parts of the manuscript. It seems to me that this is a vestige of the question not being clearly defined and things would become naturally better woven together if the question were clarified.
3. Are the data sound?
There is some mixture of "causality" type terminology with data that is observational and/or correlational.

4. Do the figures appear to be genuine, i.e. without evidence of manipulation?
NA
5. Does the manuscript adhere to the relevant standards for reporting and data deposition?
Yes
6. Are the discussion and conclusions well balanced and adequately supported by the data?
Discussion is well written, but this could be a much more powerful piece, if the question(s) were made clear.
7. Are limitations of the work clearly stated?
No – see 1st response.
8. Do the authors clearly acknowledge any work upon which they are building, both published and unpublished?
yes
9. Do the title and abstract accurately convey what has been found?
it seems that the word "health" rests mainly on the findings related to depression – suggest being more specific.
10. Is the writing acceptable?
writing style is acceptable.

Major Compulsory Revisions

Research question needs to be clarified in regards to a specific definition of empowerment (of what? For what? And why is it important?) and cannot imply any equivalence between empowerment with lack of depression—the latter is an abnormality and a serious, medical disease requiring evidence based treatment, not sociological approaches to empowerment without careful monitoring for suicidality, increasing symptoms, need for hospitalization, medication, evidence-based psychotherapy, etc. Please see response to #1 (above) for full comments on these issues.

Minor Essential Revisions
The above are more critical.
The author can be trusted to make these. For example, missing labels on figures, the wrong use of a term, spelling mistakes.

Discretionary Revisions
While it is clearly the prerogative of the writers, I think it could be quite interesting to compare empowerment needs for women before and after having children and to look at it in a comprehensive way – not only the new role as parent, but including the stress it puts on worklife, economic earnings, partnerships, etc. Across many cultures, the birth of the 1st child often marks the moment when women (versus men) halt or drastically scale back on non-parental roles in society. What does this mean about their empowerment at that time? Understandably, this could be

controversial … I think we have many good tools and models now to help women in careers, but very little about how to navigate the motherhood life change. For example – the academic studies showing the large differences in time spent on domestic duties between men and women who are at the same academic stage (K awardees) – differences that only surface after children are present. Do any parallels outside the academic worlds exist? What is the data? Is this an empowerment issue? How can we address it? So far, it has only been described in a few articles (that were massively circulated) – a great time to offer something to advance the path.

Level of interest - An article whose findings are important to those with closely related research interests

Quality of written English - Acceptable

Declaration of competing interests
I declare that I have no competing interests.

Response to reviewers

Comments from Managing Supplement Editor: Ushma Upadhyay

1. **As you will see, the reviewers' primary concerns are a need for greater clarity of the research question. Part of this may be being more specific as the scope of the interventions included in the review. Please define what you are considering to be "empowerment interventions."**

   Thank you for the opportunity to revise and resubmit our manuscript. We have carefully considered your and the two reviewers' comments, and made changes to the manuscript, as detailed below.

   We have now clarified the purpose of the paper:

   In lines 42-45 of the Abstract:
   "*Thus, the purpose of this review is to review and critically discuss the literature linking investigate if empowerment and empowerment interventions associate with reduced rates of perinatal depressive symptoms, as well as preterm birth (PTB) and low birthweight (LBW)."*

   In lines 111-113 of the Background:
   "Thus, we here *systematically review the relatively small literature that has tested the link between perinatal maternal empowerment and symptoms of perinatal depressive symptoms as well as PTB and LBW.*"

   In lines 113-119 empowerment interventions have now been defined and the scope of the studies is described:
   "For the purpose of this review, empowerment is defined broadly as a person's autonomy, decision making power and self-determination. The present review included observational studies assessing degree of empowerment or change in empowerment through questionnaires, as well as studies manipulating women's empowerment by implementing programs intended to increase women's empowerment, which we here refer to as empowerment interventions."

2. **Please also include some assessment of the quality of the included articles.**
   An assessment of the quality of the included articles, based on the NIH quality rating standards for controlled intervention studies and observational, cohort, and cross-sectional studies, was added to the tables. A Quality Ratings sub-section was also added to the Methods section.

Reviewer: Linda Franck

This manuscript presents the findings of a systematic review of the literature regarding the effects of empowerment interventions in the perinatal period on maternal depression and the incidence of preterm birth (PTB)or low birth weight (LBW) births

Major Compulsory Revisions

6. **Title: The title of the paper does not accurately reflect the content of the paper. First, it does not indicate that the paper is a systematic review of the literature. Second, it does not clearly state that the subject is empowerment interventions (rather than descriptive associations of a women's innate level of empowerment). Third, it suggests a broad range of maternal and infant health outcomes were explored, when in fact the review was restricted to three outcomes only.**
   We now include the term "systematic review" in the title. We also specified that this paper specifically addresses perinatal depressive symptoms and premature birth. As we clarify in more detail below, our review *does* include both descriptive studies and intervention studies. Therefore, we chose to continue to use the broader term "empowerment."

   The title was changed to:
   "*A Systematic Review of the Association between Perinatal Empowerment, Perinatal Depressive Symptoms, and Premature Birth*"

7. **Abstract: The abstract background is problematic as described in more detail in comment #3a-c. The term 'milestone' in line 37 is not an accurate description of the perinatal period.**
   The term 'milestone' was changed to "*time.*"
   Please see below for changes affecting #3a-c. The abstract has been modified as well.

   The term 'empowering' in line 46 is more accurately termed "empowerment interventions".
   In lines 52-53, the term 'empowering' was changed, and this section now reads:
   "*measures of empowerment as well as empowerment interventions*"

8. **Background: The Background should be revised and expanded to address the following issues:**
   a. **Empowerment and empowerment interventions during pregnancy should be defined clearly at the outset and referenced. The Background should make it obvious why the authors selected the terms in the search strategy.**

Our conceptualization of empowerment and empowerment interventions is now defined more specifically, and we adjusted our language to make it more obvious why we used the specific search terms:

In lines 113-122, we state:

"*For the purpose of this review, empowerment is defined broadly as a person's autonomy, decision making power and self-determination. The present review included observational studies assessing degree of empowerment or change in empowerment through questionnaires, as well as studies manipulating women's empowerment by implementing programs intended to increase women's empowerment, which we here refer to as empowerment interventions. More, specifically, methods of empowerment include but are not limited to promoting literacy about health monitoring, self-efficacy, self-advocacy, and self-help skills as well as education about decision making in parenting.*"

b. **Lines 69-73 are unclear and perhaps hyperbole. Please describe more precisely what "enormous and lasting" impacts on children through to society are achieved by empowerment interventions targeted at women – and what specifically are these powerful interventions and why if so powerful have they not been implemented?**

We agree. The phrase "enormous and lasting" has been removed. To clarify, we added two examples of how women's empowerment impacts children in lines 75-92:

"*Of note, the benefits of women's empowerment are not necessarily limited to the woman targeted for the intervention, but have the potential to extend to those around her including – perhaps most prominently – her own children. While the relationship between a mother and her child is likely to be close and meaningful at any age, there is perhaps no closer relationship than that between a mother and her unborn child. Stressors experienced during pregnancy not only result in physiological (e.g., adrenocortical) alterations in the pregnant woman, but these biological signals can be communicated to the unborn child via placental transmission, and have been associated with outcomes including preterm birth and postpartum depression [7].*"

Lines 78-81 are unclear. What type of empowerment interventions are the authors referring to? What about maternal anxiety, parenting self-efficacy, lifestyle or health behaviors might be impacted? The authors should explain why they have specifically focused on maternal depression and PTB/LBW?

We agree that clarification is needed. Regarding the first question we have removed irrelevant information, and regarding the second question, while there are other relevant issues, we chose to focus on

these because they are the earliest health benefits that can be assessed.
In lines 380-394, we include:
"It should be noted that this review did not aim to comprehensively review all possible maternal-infant health benefits associated with women's empowerment. Instead, it focused on one specialized aspect of this association, specifically, perinatal depressive symptoms and PTB/LBW. This is not to say that empowerment does not affect women's and children's lives in many other important ways, but rather that these are some of the earliest health benefits that can be assessed. There is ample evidence that maternal perinatal depressive symptoms continue to have negative consequences for the health and wellbeing of the mother and child. For example, associations have been shown with infant negative affectivity [10], poor mother-infant bonding [11], elevated parenting stress [12] as well as physical and mental illness [13]. Similarly, PTB and LBW have enduring adverse consequences for child health and developmental outcomes such as increased risk for neurodevelopmental disabilities [14], attention difficulties [15], and cardiovascular disease later in life [16]. Given these associations, it appears likely that perinatal empowerment is associated with other health benefits to mother and infant, some of which may also be longer lasting. While important, these studies were considered to be beyond the scope of this review"

Regarding the last question, we now explain why we specifically focused on preterm birth and perinatal depressive symptoms in lines 87-92:
"While the relationship between a mother and her child is likely to be close and meaningful at any age, there is perhaps no closer relationship than that between a mother and her unborn child. Stressors experienced during pregnancy not only result in physiological (e.g., adrenocortical) alterations in the pregnant woman, but these biological signals can be communicated to the unborn child via placental transmission, and have been associated with outcomes including preterm birth and postpartum depression [7]."
In lines 97-103:
"One of the earliest measures of infant health are measures of prematurity: preterm birth (PTB) and low birthweight (LBW); and there is convincing evidence that being born prematurely is a risk factor for poorer health outcomes throughout the life span (e.g. [8]). One early maternal health outcome is postpartum depressive symptoms. In fact, it has been argued that the biobehavioral pathways leading to PTB and postpartum depression and its symptoms may overlap [9]."

Methods:

c. **The inclusion/exclusion criteria should clarify the criteria regarding study design. The tables indicate that the authors included a wide range of study designs and yet this is not described or justified and no criteria for selection of the studies by design type is provided.**
We agree there is a need for clarification. Line 152 now states:
"No limits were set for date of publication, maternal age, *study design*, or language of publication…."

d. **The authors specify that empowerment (not defined) had to be measured or manipulated. This suggests cohort studies were included, but that is not made clear (and impacts my earlier comments about Abstract and Background).**
The definition of empowerment needed further specification. We now provide a more extensive definition of empowerment lines 113-122:
"For the purpose of this review, empowerment is defined broadly as a person's autonomy, decision making power and self-determination. The present review included observational studies assessing degree of empowerment or change in empowerment through questionnaires, as well as studies manipulating women's empowerment by implementing programs intended to increase women's empowerment, which we here refer to as empowerment interventions. More specifically, methods of empowerment include but are not limited to literacy about health monitoring, self-efficacy, self-advocacy, and self-help skills as well as education about decision making in parenting."

Cohort studies were included, and we now clarify that in line 152:
"No limits were set for date of publication, maternal age, *study design*, or language of publication; although, only English language publications were identified."

In addition, the timeframe for the intervention is not made clear. The reader is led to believe that the intervention must be delivered pre-birth in order to have an effect on depression or PTB/LBW. And yet later, the COPE intervention delivered to women with preterm/LBW babies is included.
The measure of empowerment or the empowerment intervention had to be obtained during pregnancy or within one year after delivery. We now clarify in lines 136-139:
"*To be included in this review, studies had to be peer-reviewed and include a) a sample of women who were either pregnant or within the first year post partum; b) a measure or manipulation of empowerment or the lack thereof, as defined above; and c) a measure of perinatal depressive symptoms or of prematurity (PTB, LBW)."*

The COPE intervention, which was delivered postpartum, was included because it focuses on postpartum depressive symptoms as an outcome for mothers of premature infants. To make the reader aware that the cutoff for empowerment measures/ manipulations in terms of their effect on PTB/LBW is naturally limited by birth, we also added in lines 139-142:
"Of note, measures or manipulations of empowerment implemented after birth were only relevant to the postpartum depressive symptoms outcome because they are implemented after PTB/LBW has already occurred."

The authors also specify that a health outcome must be measured, but this is not defined, where as elsewhere, the authors specifically focus on the outcomes of depression and PTB/LBW. The inclusion/exclusion criteria with regard to interventions and outcomes needs to be made more clear and specific and provide a well-reasoned justification.
We specify that the studies must include (lines 138-139):
"*a measure of perinatal depressive symptoms or of prematurity (PTB, LBW)."*
We also provide a justification in the Inclusion/Exclusion Criteria Section.
We now specify the outcomes by changing the term 'perinatal health outcome' to *"perinatal depressive symptoms or PTB and LBW"* in the Selected Studies section. The title, the abstract, and body of the document were searched and 'perinatal health outcome' was changed to specify we are referring to perinatal depressive symptoms or PTB and LBW, where relevant.

e. **Conceptualiztions of Empowerment: This section would be more accurately titled "Interventions".**
The review includes both empowerment assessments and empowerment interventions. Therefore, we chose to use the broader term "conceptualizations of empowerment."

The description of Centering Pregnancy™ is inaccurate. It is a group prenatal care model. The authors should more specifically and precisely describe the empowerment elements of the intervention and provide citations.
This has been modified to specify that this is a *group prenatal care model* (lines 189-190), and that it was included in the present review because *it is aims to empower women by increasing their health self-care efficacy during pregnancy* (line 190-191). A citation of Rising (1998) has been provided.

As noted in 4b, COPE seems an inappropriate intervention to include, given that it is initiated after PTB/LBW.
We agree that this needs clarification. Creating Opportunities for Parent Empowerment was included because, although it is delivered in the postpartum period, it may potentially reduce the risk for maternal postpartum depressive symptoms by increasing mothers' parenting self-efficacy with their premature infant. One of the intended outcomes is decreases in parenting anxiety and postpartum depressive symptoms for mothers of premature infants. This is now specified and clarified in the Inclusion and Exclusion Criteria section.

Moreover, the empowerment elements are not clearly stated and referenced. Similar problems with definitions and time points for interventions and outcomes measurement are noted in interventions described in lines 135-145.
Citations for the interventions have now been provided and information about time points of interventions and outcomes are included in the tables.

It appears conceptually inconsistent to include prenatal and postnatal interventions for depression and PTB/LBW in the same review.
We agree that this is conceptually inconsistent; however, it is practically the case that perinatal depressive symptoms can begin after delivery. We carefully weighed the benefits of removing the studies that conducted empowerment assessments post partum, but felt that the downside of this approach is that we are omitting some of the relevant literature. In weighing the potential downsides of either approach, we finally decided to keep the studies in the review for reasons of being comprehensive. We provided clarification in the methods section about this choice that may be seen as incongruous by readers.
In lines 139-142, we include:
"Of note, measures or manipulations of empowerment implemented after birth were only relevant to the postpartum depressive symptoms outcome because they are implemented after PTB/LBW has already occurred."

f. **No quality assessment of the studies is provided and this should be done.**
We agree. A quality assessment of the studies has been conducted according to NIH quality assessment standards, and the tables now include quality rating scores. A Quality Ratings sub-section was also added to the Method section.

g. **Although the authors reference PRISMA, it does not appear that they followed the PRISMA (or any other systematic review guidelines) for their selection of studies and analyses. The paper would be greatly strengthened by following such guidelines.**
   To the best of our knowledge, we followed PRISMA standards, and a flowchart is included (Figure 1).

9. **Results/Discussion: Because of the major methodological and conceptual concerns described above, the reviewer cannot adequately comment on the Results and Discussion. Moreover, previous systematic reviews of Centering Pregnancy™ and COPE™ have not been included in the discussion.**
   Additional clarification of methodological and conceptual items was needed and has now been provided. Previous systematic reviews of CP and COPE had not been included because they did not focus on elements of empowerment.

Reviewer: Susan Meffert

1. Is the question posed by the authors well defined?
While I agree with the authors that the transition from woman to mother (especially with the 1st child), poses a substantial risk to empowerment, however it is defined, I am not sure that they authors clearly define terms beyond that. Specifically, there seem to be a number of assumptions embedded within the research question that should be more clearly defined. First, as many of the empowerment interventions that are discussed in the paper revolve around helping women to care for themselves as mothers and to teach parenting/childcare skills, there seems to be an assumption that the difficulties women experience with empowerment after having a child revolve around their struggle to be mothers. While parenthood is not to be underestimated, in my work, I have observed that the struggle to remain empowered in work outside the home and as an economically productive individual is quite high stakes for new mothers – for example, in our studies with Kenya HIV+ women, having a child often marks the a halt of work outside the home beginning of a period of higher economic dependency on husbands, reducing empowerment and sometimes leading to gender based violence.

We agree. There are many other ways in which women struggle to remain empowered in the perinatal period. The focus on parenthood is a limitation resulting from the content of the small body of literature found during our search. Information about this has now been included in the Discussion section.

In lines427-439, we include:
"*Moreover, we note that existing studies have focused on empowerment as it specifically relates to women's parental role. While the parental role is perhaps most salient in the context of empowerment of pregnant women and new mothers, other significant life changes occur with the birth of a child, in particular the birth of a first*

child. Across many cultures, new mothers often scale back on or halt involvement in non-parental societal roles such as work outside the home, leading to decreases in financial independence as well as changes in social relationships and social status. We could not identify any studies that tested whether the empowerment of pregnant or postpartum women in domains other than the parental one is associated with perinatal depressive symptoms or prematurity. It would be an important contribution to the literature to study the relative impact of different facets of empowerment during the perinatal period on maternal-infant birth outcomes, because this knowledge would provide the groundwork for targeted interventions.

One way around this would be to be very specific that you want only to investigate strategies for empowering new mothers in their roles as parents. Then, you would need a very good justification for why that is a key issue on which to focus.

We agree. Particularly, the studies assessing postpartum depressive symptoms as an outcome, largely focus on strategies for empowering new mothers in their roles as parents, in caring for premature infants. We now clarify in the discussion section that our search, while broad, only yielded manuscripts that addressed empowering new mothers in their role as parents. In line with the reviewer's suggestion, we propose that other areas of change in women's lives might also be important to address.

Lines 427-439, state:

"Second, we note that existing studies have focused on empowerment as it specifically relates to women's parental role. While the parental role is perhaps most salient in the context of empowerment of pregnant women and new mothers, other significant life changes occur with the birth of a child, in particular in the context of the birth of a first child. Across many cultures, with the birth of a first child, women often scale back or halt involvement in non-parental societal roles such as work outside the home leading to decreases in financial independence, and to changes in social relationships and social status. We did not identify any studies that tested whether the empowerment of pregnant or postpartum women in domains other than the parental one is associated with perinatal depressive symptoms or prematurity. It would be an important contribution to the literature to study the relative impact of different facets of empowerment during the perinatal period on maternal-infant birth outcomes, because this knowledge would provide the groundwork for targeted interventions."

My other concern about the specification of the question is that there seems to be conflation of the terms depression, empowerment, overall health and even PTSD. Depression and PTSD are medical diseases and are not normal responses to stress—even that as severe as first time parenthood. While empowerment is certainly important, it is not an evidence-based treatment for depression or PTSD and it would be misleading (and potentially damaging) to suggest that it is. My suggestion would be either focus VERY specifically on depression, acknowledging nad defining it as the medical disease it is, then explain why the relationship between empowerment and depression is

important even though empowerment is not an evidence based treatment for depression (e.g., you might look at risk factors/prevention).

We agree with the reviewer's suggestion, which we think may have been, at least in part, driven by the imprecise language we used in parts of our previous submission. Throughout the manuscript (including the title, abstract and text), we now use the more appropriate term "*postpartum depressive symptoms*", which does not imply a medical disease. In terms of PTSD, line 255 now indicates that we are referring to "*symptoms indicative of PTSD risk.*"

2. Are the methods appropriate and well described?
Yes, although I would suggest re-organization such that there are not two distinct parts of the manuscript. It seems to me that this is a vestige of the question not being clearly defined and things would become naturally better woven together if the question were clarified.

We hope that the clarification of the purpose of our review, which we describe in more detail in response to the reviewer's other comments, also appropriately address the reviewer's concern regarding the two somewhat distinct parts of the manuscript.

To further clarify, we now also give a reason for presenting the results for the two sets of outcomes separately at the beginning of our results section. Lines 214-216 now read:

"No studies were identified that addressed both maternal and infant outcomes, and we therefore report on the two types of outcomes separately."

In the discussion section, we further point out that the literature on perinatal depressive symptoms and PTB/LBW remains distinct (paragraph starting in line 380) and we provide thoughts on why integrative studies hold merit.

Finally, one strength of the decision to keep these sections separate is that they may be of interest to a different subset of readers, in particular those with an interest in child development (birth outcomes) versus women's health interests (postpartum depressive symptoms).

3. Are the data sound?
There is some mixture of "causality" type terminology with data that is observational and/or correlational.

Thank you for pointing out our use of causal language. We carefully read through the manuscript to remove all causal language and replace it with terms that more appropriately describe correlational associations.

4. Do the figures appear to be genuine, i.e. without evidence of manipulation?

NA
5. Does the manuscript adhere to the relevant standards for reporting and data deposition?
Yes
6. Are the discussion and conclusions well balanced and adequately supported by the data?
Discussion is well written, but this could be a much more powerful piece, if the question(s) were made clear.
We hope that the revisions we made to the discussion section, further improved its quality. The research questions have now been clarified. The purpose of the review is clarified in the Background section.

7. Are limitations of the work clearly stated?
No – see 1st response.
We now discuss the limitations in the current literature and how those could be addressed in a section on Recommendations for Future Work (starting line 399).

8. Do the authors clearly acknowledge any work upon which they are building, both published and unpublished?
yes
9. Do the title and abstract accurately convey what has been found?
it seems that the word "health" rests mainly on the findings related to depression – suggest being more specific.
We have made our language in the title and abstract more precise. The title has been modified to:
"A Systematic Review of the Association between Perinatal Empowerment, Perinatal Depressive Symptoms, and Premature Birth"

Additionally, we removed the broad term "health" and replaced it with the more specific terms perinatal depressive symptoms and PTB or LBW.

10. Is the writing acceptable?
writing style is acceptable.

Reviewer's report

- Major Compulsory Revisions

The author must respond to these before a decision on publication can be reached. For example, additional necessary experiments or controls, statistical mistakes, errors in interpretation.

1. **Research question needs to be clarified in regards to a specific definition of empowerment (of what? For what? And why is it important?) and cannot imply any equivalence between empowerment with lack of depression—the latter is an abnormality and a serious,**

medical disease requiring evidence based treatment, not sociological approaches to empowerment without careful monitoring for suicidality, increasing symptoms, need for hospitalization, medication, evidence-based psychotherapy, etc. Please see response to #1 (above) for full comments on these issues.
The purpose of the study has now been clarified in the Background section, and we now clarify that empowerment is a sociological approach and not a treatment for depression. Additionally, we have replaced language that implies the medical disease, for language that more accurately reflects that we are referring to depressive symptoms.

2.

- Minor Essential Revisions
The above are more critical.
The author can be trusted to make these. For example, missing labels on figures, the wrong use of a term, spelling mistakes.

We carefully proofread the manuscript to fix any remaining editorial issues we could identify.

- Discretionary Revisions

3. **While it is clearly the prerogative of the writers, I think it could be quite interesting to compare empowerment needs for women before and after having children and to look at it in a comprehensive way – not only the new role as parent, but including the stress it puts on worklife, economic earnings, partnerships, etc. Across many cultures, the birth of the 1st child often marks the moment when women (versus men) halt or drastically scale back on non-parental roles in society. What does this mean about their empowerment at that time? Understandably, this could be controversial ... I think we have many good tools and models now to help women in careers, but very little about how to navigate the motherhood life change. For example – the academic studies showing the large differences in time spent on domestic duties between men and women who are at the same academic stage (K awardees) – differences that only surface after children are present. Do any parallels outside the academic worlds exist? What is the data? Is this an empowerment issue? How can we address it? So far, it has only been described in a few articles (that were massively circulated) – a great time to offer something to advance the path.**
Thanks for sharing this insight. We agree that, particularly, with the birth of the first child, there is a drastic change in work outside of home and other activities not associated with the parental role. We recognize the importance of these issues, and now include them in the Discussion section, under the Recommendations for Future Work subsection.

In lines 427-439:

"Moreover, we note that existing studies have focused on empowerment as it specifically relates to women's parental role. While the parental role is perhaps most salient in the context of empowerment of pregnant women and new mothers, other significant life changes occur with the birth of a child, in particular the birth of a first child. Across many cultures, new mothers often scale back on or halt involvement in non-parental societal roles such as work outside the home, leading to decreases in financial independence as well as changes in social relationships and social status. We could not identify any studies that tested whether the empowerment of pregnant or postpartum women in domains other than the parental one is associated with perinatal depressive symptoms or prematurity. It would be an important contribution to the literature to study the relative impact of different facets of empowerment during the perinatal period on maternal-infant birth outcomes, because this knowledge would provide the groundwork for targeted interventions."

Review reports – 2nd round

Reviewer 1: Linda Franck

The authors are to be commended for their efforts and detailed revisions and response to reviewers' concerns. For me, some significant fundamental concerns remain. I do not think this is properly conceived. They lump empowerment measurement and 'manipulation' together and that leads them to make false conclusions about the state of the science. The inclusion criteria should be: a) a direct measure of empowerment (descriptive studies) and b) a direct measure of empowerment AND an intervention aimed at increasing empowerment. Many of the included studies do not conform to criteria b.

-Definition of the perinatal period and inclusion of the first year of life in the review. It is my continued belief that these are different enough to be separated out. I do not think that the authors have provided adequate justification for inclusion. Perhaps they could present and analyze separately?
-I remain concerned as to how they describe Centering Pregnancy. Moreover – I do not think that the authors of CP claim that it's mechanism of action is solely through Empowerment. I have huge concerns that the authors of this review see any study that had an empowerment component as being eligible for inclusion, even if empowerment was never directly measured – as n the CP studies.

Other editorial issues:
I started to try to address some of the concerns by suggesting alternate wording – I stopped when I got to the results
-In particular, use of phrases that suggest empowerment is something done to women is problematic, rather than it being an internal state that can be facilitated or supported; The authors persist in using language that states or implies that a person can be made to be empowered; and then contradict themselves by describing empowerment as an internal state that could be enhanced, facilitated or supported. Please encourage them to be consistent and precise.

I simply cannot in good conscience recommend this paper for publication in its current form. I'm sorry I can't give a more positive review. Kind regards, Linda

Reviewer 2: Susan Meffert
No additional questions -- the revisions look fine to me.

Response to reviewers – 2nd round

Thank you for the opportunity to submit a revised manuscript. We were pleased to see that our previous revision was satisfactory to the second reviewer, and we appreciate the thoughtful additional comments provided by reviewer 1. We considered all remaining concerns very carefully and are confident that our changes resulted in a strengthened manuscript, which we hope is now suitable for publication.

Reviewer: Linda Franck

1. The authors are to be commended for their efforts and detailed revisions and response to reviewers' concerns. For me, some significant fundamental concerns remain. I do not think this is properly conceived. They lump empowerment measurement and 'manipulation' together and that leads them to make false conclusions about the state of the science. The inclusion criteria should be: a) a direct measure of empowerment (descriptive studies) and b) a direct measure of empowerment AND an intervention aimed at increasing empowerment. Many of the included studies do not conform to criteria b.

We understand the concern. The concern with including criterion b is that there are no studies that had both an intervention AND a direct measure of empowerment. We struggled with the idea of taking the bulk of the studies out of the review, because we continue to feel that they make an important contribution to the literature, and also create awareness of the fact that intervention studies measuring empowerment directly are lacking in this area of research.

We therefore decided to make clear, for each study, how empowerment was measured or conceptualized, and now detail this information in Table 3.

We also included the following text in the Method section of our manuscript to clarify this for the reader:

> *"Of note, we could not identify studies that implemented empowerment interventions and also obtained measures of empowerment. Moreover, the interventions in the studies reported here were not always designed to improve a health outcome by changes in empowerment alone. Table 3 shows for each study which measure or intervention was used, and how empowerment was conceptualized in the context of that particular study."*

In the discussion section, we further highlight this problem as a limitation in the literature and point toward the need to conduct intervention studies that measure changes in empowerment directly.

> *"It should also be noted that empowerment was not measured directly in any of the intervention studies presented here. All interventions included at least an element of empowerment (see Table 3 for details), and it seems likely that supporting women's empowerment was a contributor to the health benefits observed. Nonetheless, because empowerment was not directly measured, it*

cannot be concluded with confidence that it was, in fact, a change in empowerment that lead to the observed health benefits. In fact, it cannot be determined whether the intervention was even accompanied by a change in women's perception of empowerment. There is a need for studies that test whether empowerment is indeed a pathway through which these interventions contribute to improving maternal infant health outcomes. We recommend that future intervention studies administer a direct measure of empowerment before and after the intervention."

2. Definition of the perinatal period and inclusion of the first year of life in the review. It is my continued belief that these are different enough to be separated out. I do not think that the authors have provided adequate justification for inclusion. Perhaps they could present and analyze separately?

We see the point. We considered separating the tables, but found it problematic for those studies that included assessments of depressive symptoms during pregnancy and post partum. Instead, we opted to highlight this information in columns 4 and 5 of both tables. For each study we now indicate specifically whether a measure was obtained in pregnancy (PREG) or post partum (PP). A sentence was added in the methods section stating how many articles assessed empowerment and depression in pregnancy, postpartum, or both:

In line 152:

> *"We identified one study assessing depressive symptoms during pregnancy, 11 in the postpartum period, and four in both pregnancy and postpartum. Empowerment was measured or an intervention supporting empowerment was administered in the postpartum for nine studies, in pregnancy for three studies, and in both for four studies."*

We also provide some information about this issue in the results section, differentiating by time point in which depressive symptoms were measured.

In line 239:

> *"There were no observable differences in study outcomes depending on when depressive symptoms were assessed. When assessed in the post partum, nine studies found significant associations, and two did not. The only study that assessed depressive symptoms in pregnancy found significance. Finally, of the studies that assessed depressive symptoms in both pregnancy and postpartum, two found significance and two did not."*

To add justification for inclusion of the first postpartum year for studies on postpartum depression, we now point interested reviewers to our recent review which shows that available studies of risk factors for postpartum depression have conducted assessments of postpartum depression anywhere between 1 day and 1 year postpartum. This sentence now reads:

> *"In terms of maternal health, one early birth outcome is the presence and degree of postpartum depressive symptoms, which in the broader postpartum depression*

literature has been assessed anywhere between 1 day and 1 year post partum [10].

3. I remain concerned as to how they describe Centering Pregnancy. Moreover – I do not think that the authors of CP claim that it's mechanism of action is solely through Empowerment. I have huge concerns that the authors of this review see any study that had an empowerment component as being eligible for inclusion, even if empowerment was never directly measured – as n the CP studies.
We understand the concern, and have provided further clarification. CP's mechanisms of action include, but are not limited to empowerment. We have added quotes from Rising (1998), which describe CP and its empowerment components, and we have further described the elements of empowerment in Table 3.
Lines 190-192:

> *"Centering Pregnancy unifies the components of prenatal care—risk assessment, education, and support within the group—and encourages women to take responsibility for their own health [20, p. 46]."*
> Lines 195-198:
> *"The woman's involvement in self-care activities, the discussion and education format, the worksheets and handouts, and the sharing among the women all lead to her enhanced sense of empowerment. This, in turn, results in a sharing of power between the provider and the consumer [20, p. 53]."*

We believe that the changes we made in response to this Reviewer's comment 1 also helped to address this issue.

4. Other editorial issues:
I started to try to address some of the concerns by suggesting alternate wording – I stopped when I got to the results
Thanks for the suggestions, we have taken them into consideration and have modified the wording accordingly.

5. In particular, use of phrases that suggest empowerment is something done to women is problematic, rather than it being an internal state that can be facilitated or supported; The authors persist in using language that states or implies that a person can be made to be empowered; and then contradict themselves by describing empowerment as an internal state that could be enhanced, facilitated or supported. Please encourage them to be consistent and precise.
We agree that empowerment is an internal state that can be facilitated or supported. Where relevant, we have changed language to state that empowerment is an internal state that can be enhanced, facilitated or supported.

I simply cannot in good conscience recommend this paper for publication in its current form. I'm sorry I can't give a more positive review. Kind regards, Linda
We are very grateful for the depth of comments you have provided to us, and we hope that the additional changes we made may change your opinion and recommendation for

publication. Below, we have a few more responses to the comments you made directly in the manuscript. Thanks a lot, Esmeralda and Ilona.

<u>Response to comments on the document:</u>

1. define the perinatal period

In line 38 we define:

> *"The perinatal period, which we here define as pregnancy and the first year postpartum…"*

2 and 4. Change to Supporting Empowerment. Empowerment is not done to women.

We have changed *"empowering women"* to *"supporting women's empowerment"* here and in other places, where relevant.

3. This contradicts your last sentence above. It seems to me your conclusion is that it remains unclear if….

We have changed the statement to be more accurate.

Line 59 now reads:

> *"This small body of work suggests, for the most part, that empowerment may be protective for perinatal depressive symptoms and PTB/ LBW."*

5. When? For how long?

We agree that clarification is needed, and now state in line 102:

> *"In terms of maternal health, one early birth outcome is the presence and degree of postpartum depressive symptoms, which in the broader postpartum depression literature has been assessed anywhere between 1 day and 1 year post partum [10]."*

We also clarified in the abstract:

> *"The perinatal period, which we here define as pregnancy and the first year post partum, …"*

6. Please delete [manipulating women's empowerment]

We've deleted the phrase, in line 117, and substitute it with
"supporting women's empowerment"

We have also made this change throughout the manuscript, as applicable.

7. Please reword to more simply add that these search terms were added based on their presence in initial results- otherwise it looks like you are giving results/endorsing interventions already.

We agree that this may benefit from being worded more simply.

Lines 136-138 are modified to state:

"The search terms Centering Pregnancy, child marriage, and sexual activity also emerged as relevant due to their presence in the initial search results, and the three terms were thus added to the final search."

8. This has not yet been justified in the background [within the first year postpartum]

To address this, we have included, in line 104:
"In the broader postpartum depression literature, assessments of postpartum depressive symptoms are conducted anywhere between 1 day and 1 year postpartum [10]."

9. Please use intervention rather than manipulation throughout.

We conducted a thorough search of the paper and changed "manipulation" to "intervention", where relevant.

10. This is not accurate [Centering Pregnancy (CP), an educational, support, and risk assessment group intervention prenatal care model..]

In response to the concern of an inaccurate portrayal of Centering Pregnancy, we refer back to the paper by Rising (1998). To alleviate any concerns, we now use direct quotes from the paper.

Lines 190-192:
"Centering Pregnancy unifies the components of prenatal care—risk assessment, education, and support within the group—and encourages women to take responsibility for their own health [20, p. 46]."

Lines 195-198:
"The woman's involvement in self-care activities, the discussion and education format, the worksheets and handouts, and the sharing among the women all lead to her enhanced sense of empowerment. This, in turn, results in a sharing of power between the provider and the consumer [20, p. 53]."

Reviewer: Susan Meffert
No additional questions -- the revisions look fine to me.
